# Cytotoxic and Antifungal Constituents Isolated from the Metabolites of Endophytic Fungus DO14 from *Dendrobium officinale*

**DOI:** 10.3390/molecules21010014

**Published:** 2015-12-22

**Authors:** Ling-Shang Wu, Min Jia, Ling Chen, Bo Zhu, Hong-Xiu Dong, Jin-Ping Si, Wei Peng, Ting Han

**Affiliations:** 1 Nurturing Station for the State Key Laboratory of Subtropical Silviculture, Zhejiang A & F University, Lin’an 311300, China; shang2002012@163.com (L.-S.W.); aurora0119@163.com (B.Z.); dhx0103@126.com (H.-X.D.); lssjp@163.com (J.-P.S.); 2 Department of Pharmacognosy, School of Pharmacy, Second Military Medical University, Shanghai 200433, China; jm7.1@163.com (M.J.); m15201916813@163.com (L.C.); 3 College of Pharmacy, Chengdu University of Traditional Chinese Medicine, Chengdu 610075, China

**Keywords:** *Dendrobium officinale*, endophytic fungi, cytotoxic activities, antifungal activities, metabolites

## Abstract

Two novel cytotoxic and antifungal constituents, (4*S*,6*S*)-6-[(1*S*,2*R*)-1, 2-dihydroxybutyl]-4-hydroxy-4-methoxytetrahydro-2*H*-pyran-2-one (**1**), (6*S*,2*E*)-6-hydroxy-3-methoxy-5-oxodec-2-enoic acid (**2**), together with three known compounds, LL-P880γ (**3**), LL-P880α (**4**), and Ergosta-5,7,22-trien-3b-ol (**5**) were isolated from the metabolites of endophytic fungi from *Dendrobium officinale*. The chemical structures were determined based on spectroscopic methods. All the isolated compounds **1**–**5** were evaluated by cytotoxicity and antifungal effects. Our present results indicated that compounds **1**–**4** showed notable anti-fungal activities (minimal inhibitory concentration (MIC) ≤ 50 μg/mL) for all the tested pathogens including *Candida albicans*, *Cryptococcus neoformans*, *Trichophyton rubrum*, *Aspergillus fumigatus*. In addition, compounds **1**–**4** possessed notable cytotoxcities against human cancer cell lines of HL-60 cells with the IC_50_ values of below 100 μM. Besides, compounds **1**, **2**, **4** and **5** showed strong cytotoxities on the LOVO cell line with the IC_50_ values were lower than 100 μM. In conclusion, our study suggested that endophytic fungi of *D. officinale* are great potential resources to discover novel agents for preventing or treating pathogens and tumors.

## 1. Introduction

Since the endophytic fungus which can produce taxol was reported by Stroble *et al.* [1], finding the suitable endophytic fungi to ferment and synthesize extensive active constituents has been considered as one of the effective way to resolve the resource shortage of some plant-derived compounds [2]. Endophytic fungi, microorganisms that reside in tissues of the host plant and can cause no apparent harm to the host plant during a certain phase in their life cycle [3], are known to produce some rare and novel natural agents with notable pharmacological activities including anti-tumor and anti-microbial, *etc.* [4]. However, only very few of them have been cultivated and screened for drugs.

*Dendrobium officinale* Kimura et Migo, is ranked “the first of the nine Chinese fairy herbs”, which has been officially recorded in Chinese pharmacopoeia (Pharmacopoeia Committee of the People’s Republic of China 2010). *D. officinale* possesses great medicinal values for maintaining tonicity of stomach, promoting the body fluid production, reducing peripheral vascular obstruction, preventing the development of cataracts and enhancing the immune system, and has been commonly applied to anti-tumor, anti-aging, regulation of blood sugar, treatment of stomach disorders, *etc*. [5]. However, it has been listed as an endangered species and catalogued in the Chinese Plant Red Book since 1987 because limited natural resources and high demand threaten the survival of the species [6]. Therefore, protecting the wild resources of *D. officinale* becomes increasingly important for China. As a representative species of Orchidaceae, fungi are reported to play a critical role for seed germination and plants survival of *D. officinale* [7]. Therefore, exploiting the endophytic fungi in *D. officinale* is necessary, which can not only provide fungal resources for screening potential natural products but also lay a foundation of the further study on endophyte-host interaction. However, for endophytic fungi associated with medicinal orchids, especially in the *Dendrobium* genus, only a few have been explored.

In the course of our continuous search for plant–fungus associations and novel bioactive secondary metabolites [8] from endophyte cultures, we selected a fungus *Pestalotiopsis* sp. DO14 that can increase the content of main medicinal compounds (e.g., polysaccharides) of *D. officinale* from the shoot of *D. officinale* plants collected in Yandang Mountain, Zhejiang Province, People’s Republic of China (unpublished). Through bioassay-oriented fractionation, two new monoterpenoids, (4*S*,6*S*)-6-[(1*S*,2*R*)-1,2-dihydroxypentyl]-4-hydroxy-4-methoxytetrahydro-2*H*-pyran-2-one (**1**) and (6*S*,2*E*)-6-hydroxy-3-methoxy-5-oxodec-2-enoic acid (**2**), together with three known compounds, LL-P880γ (**3**), LL-P880α (**4**) and Ergosta-5,7,22-trien-3β-ol (**5**), were isolated from the culture broth of *Pestalotiopsis* sp. DO14. We report herein the details of the isolation and identification of endophytes and compounds, and the evaluation for cytotoxic and antifungal activity of those isolated compounds.

## 2. Results and Discussion

### 2.1. Identification of the Endophytic Fungus

**Figure 1:**
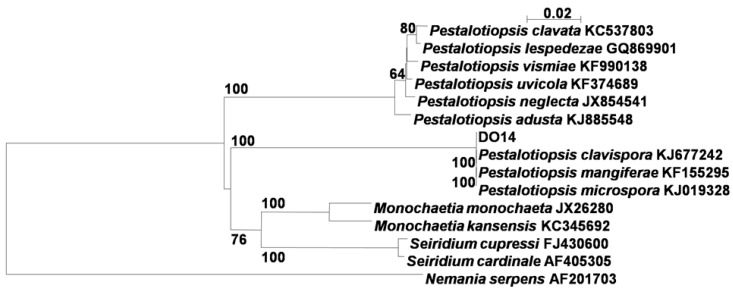
Phylogenetic tree of the endophytic fungus DO14 based on 5.8*S* and ITS regions sequences. Bootstrap values above 50% (1000 replicates) are shown at branches. *Nemania serpens* is used as an out-group.

The phylogenetic tree (Figure 1) inferred from the ribosomal DNA ITS (Internal Transcribed Spacer) sequences indicated that the endophytic fungus DO14 was classified into the clade including *Pestalotiopsis clavispora* KJ677242, *P. mangiferae* KF155295, *P. microspora* KJ019328. Thus, the endophytic fungus DO14 was identified as a *Pestalotiopsis* sp. closely related to these three taxa with the ITS sequence similarity of 100.0%.

### 2.2. Structural Determination of the Compounds

**Figure 2:**
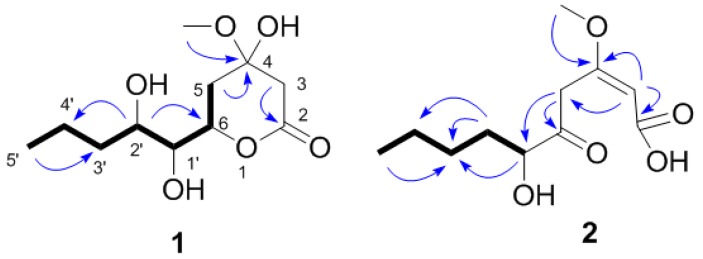
Selected ^1^H–^1^H COSY (━) and HMBC (→) correlations of **1** and **2**.

**Figure 3:**
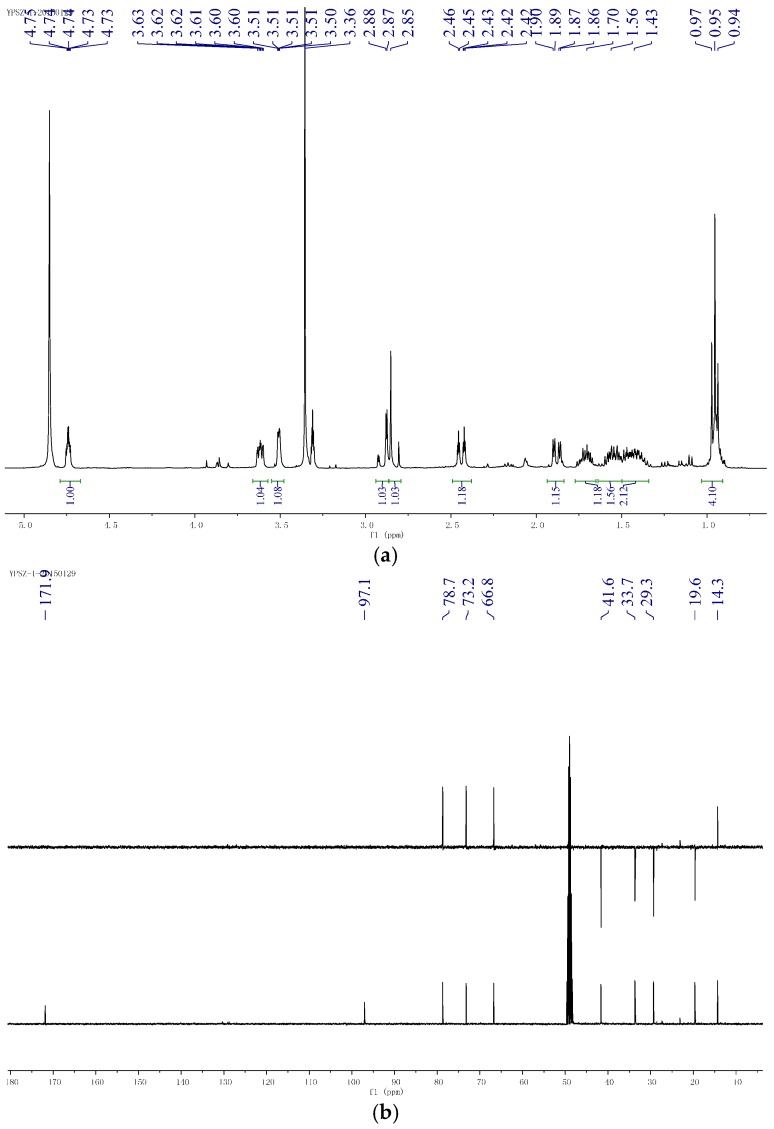

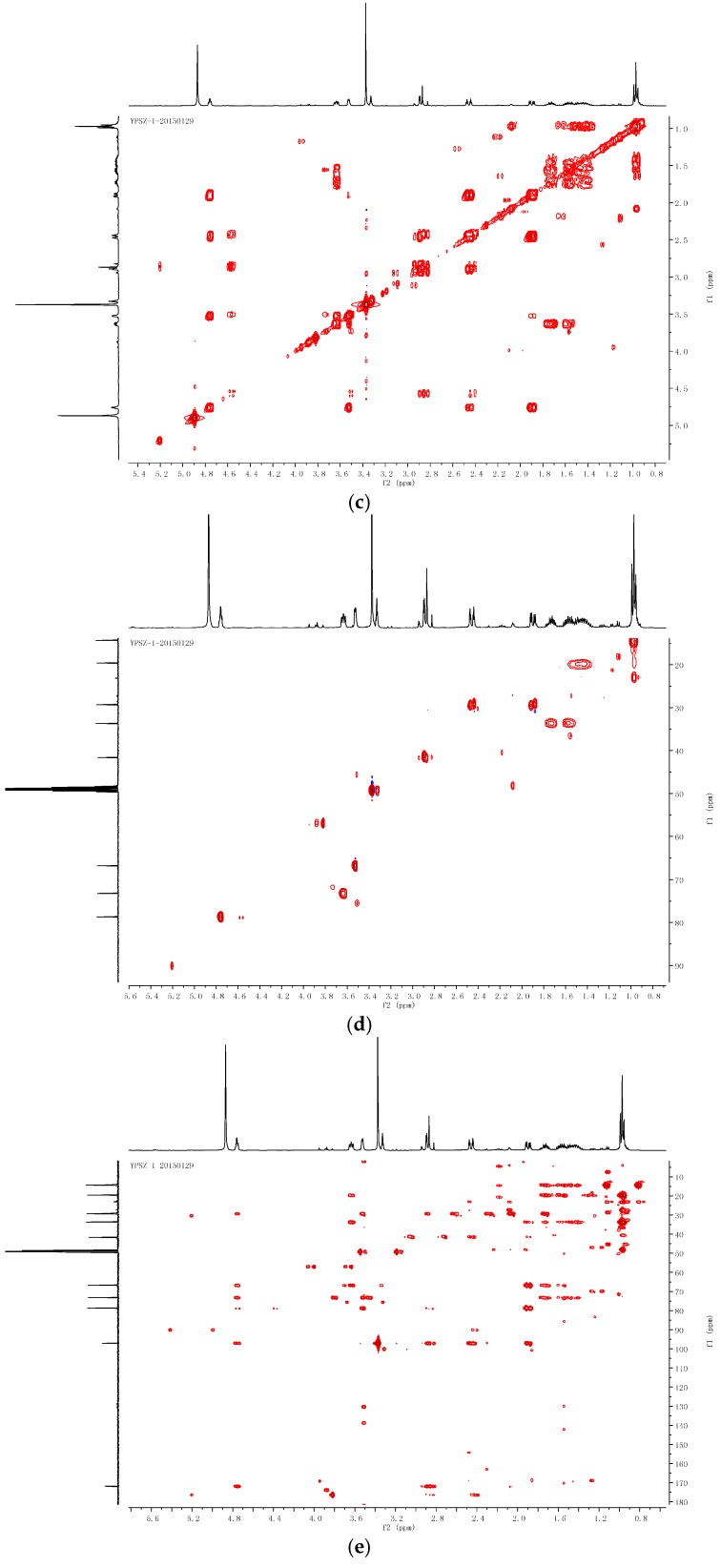

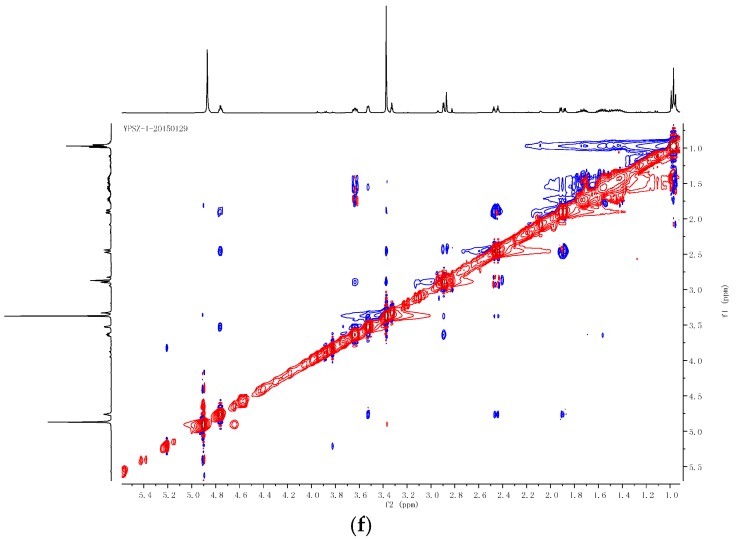
Supporting information of compound **1**. (**a**) ^1^H-NMR of compound **1**; (**b**) ^13^C-NMR and DEPT (Distortionless Enhancement by Polarization Transfer) of compound **1**; (**c**) HSQC (Heteronuclear Multiple-Quantum Correlation) of compound **1**; (**d**) ^1^H-^1^H COSY (correlated spectroscopy) of compound **1**; (**e**) HMBC (Heteronuclear Multiple Bond Correlation) of compound **1**; (**f**) NOESY of compound **1**.

**Figure 4:**
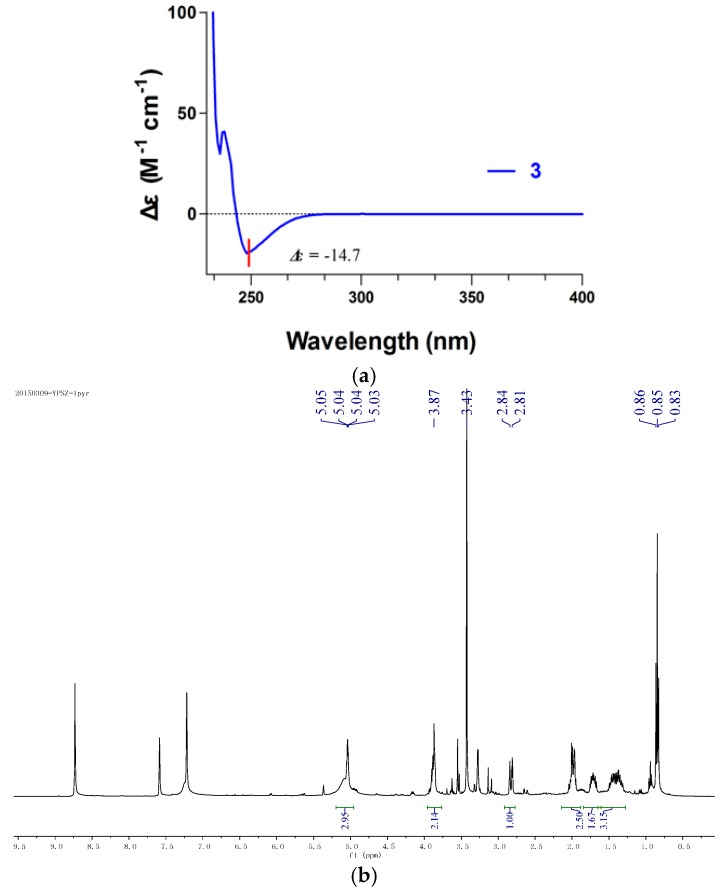

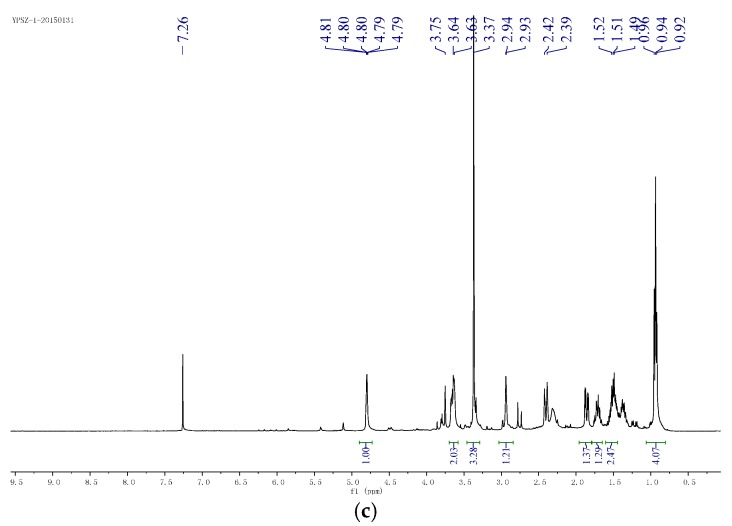
Absolute configuration of compound **1**. (**a**) CD spectrum of compound **3** in MeOH; (**b**) ^1^H-NMR spectrum of compound **1** in C_5_D_5_N; (**c**) ^1^H-NMR spectrum of compound **1** in CDCl_3_.

Compound **1** was obtained as yellow oil (${\left[\alpha \right]}_{\text{D}}^{25}$ −14.3 (*c* 0.50, MeOH) ) and analyzed for the molecular formula C_11_H_20_O_6_ by HRESIMS [M − H]^−^ at *m*/*z* 247.1186 (cald. 247.1182). The IR spectrum exhibited absorption bands for hydroxyl groups (3406 cm^−1^) and carbonyl groups (1713 cm^−1^). The ^1^H-NMR spectra data exhibited signals for one-methyl groups (δ_H_ H 0.95, 3H, t, *J* = 7.3 Hz) and oxymethyl (δ_H_ H 3.36, 3H, s) (Table 1). The ^13^C-NMR spectrum together with DEPT data resolved 11 carbon resonances attributable to one carbonyl group (δ_c_ C 171.9), two methyls, three sp^3^ oxygenated methines, four sp^3^ methylenes, and one sp^3^ oxygenated quaternary carbons (Table 1). As one of the two degrees of unsaturation was consumed by one carbonyl group, the remaining degree of unsaturation required that compound **1** was monocyclic. The above-mentioned information was quite similar to that of co-isolated compound **3** reported from the same genus [9]. In comparison with compound **3**, the major differences of compound **1** were due to an additional oxygenated quaternary carbon (δ_c_ C 97.1) and one sp^3^ methylene (δ_c_ C 41.6) instead of one tri-substituted double bonds (δ_c_ C 173.4 and 89.7), indicating that compound **1** was a derivative of compound **3**. HMBC correlations from H_3_CO-4 (δ_H_ H 3.36) to C-4 (δ_c_ C 97.1) and from H_2_-3 (δ_H_ H 2.85 and 2.82) to C-4, C-2 and C-5 assigned that the hydroxyl was connected at C-4 (Figure 2). The planar structure of compound **1** was further established by detailed interpretation of its 2D NMR data (Figure 3). The relative configuration of compound **1** was established by comparison of 1D NMR data with compound **3**. The absolute configuration of compound **3** was confirmed by exciton chirality method in previous work [10]. The stereochemistry of compound **3** was resolved by comparing optical rotation ${\left[\alpha \right]}_{\text{D}}^{25}$ −44.3 (*c* 1.30, MeOH) and the CD (Circular dichroism spectra) curve of compound **3** shows the cotton effects at 248 nm (Δε = −14.7), indicating that the configurations of C-6, C-1′, and C-2′ were *S*, *S*, and *R*. (Figure 4A). The absolute configuration of compound **1** was confirmed by the chemical correlation from compound **3** to compound **1** in methanol dropping with H_2_O_2_ and NaOH at room temperature. Therefore, the configurations of C-6, C-1′, and C-2′ in **1** were the same as **3**. As no convincing evidence was observed in the NOESY (nuclear Overhauser enhancement spectroscopy) spectrum to assign the configuration of 4-OH, the ^1^H-NMR data of **1** was measured in CDCl_3_ and C_5_D_5_N to obtain the pyridine-induced solvent shifts [11] (Figure 4B,C). The solvent shifts of H-6 (ΔδCDCl_3_ − C_5_D_5_N = 0.25) indicated that the 4-OH/H-6 was compound **1**, 3-diaxial-oriented. Thus, 4-OH was assigned in α-orientation and the configurations of C-4 was *S*. Therefore, compound **1** was determined to be (4*S*,6*S*)-6-[(1*S*,2*R*)-1,2- dihydroxypentyl]-4-hydroxy-4-methoxytetrahydro-2*H*-pyran-2-one.

**Table 1:** NMR data of compounds **1**, **2**.

	1 ^a^		2 ^b^	
	δ_C_, Type	δ_H_ Mult (*J* in Hz)	δ_C_	δ_H_ Mult (*J* in Hz)
2	171.9, C		184.4, C	
3	41.6, CH_2_	2.88, dd (18.5, 2.3)	105.8, CH	5.63, s
		2.85, d (18.5)		
4	97.1, C		167.1, C	
5	29.3, CH_2_	2.44, dt (13.5, 2.3)	36.5, CH_2_	3.55, 2H, s
		1.88, dd (13.5, 4.1)		
6	78.7, CH	4.74, m	204.6, C	
1′	66.8, CH	3.51, m	86.7, C	4.46, dd (7.8, 4.2)
2′	73.2, CH	3.61, m	30.7, CH_2_	1.88, m
				1.70, m
3′	33.7, CH_2_	1.70, m	26.5, CH_2_	1.40, 2H, m
		1.56, m		
4′	19.6, CH_2_	1.43, 2H, m	22.3, CH	1.34, 2H, m
5′	14.3, CH_3_	0.95, t (7.3)	13.8, CH_3_	0.89, t (7.3)
CH_3_O-4	49.2, CH_3_	3.36, s	52.6, CH_3_	3.76, s

**^a^** in CD_3_OD. **^b^** in CDCl.

**Figure 5:**
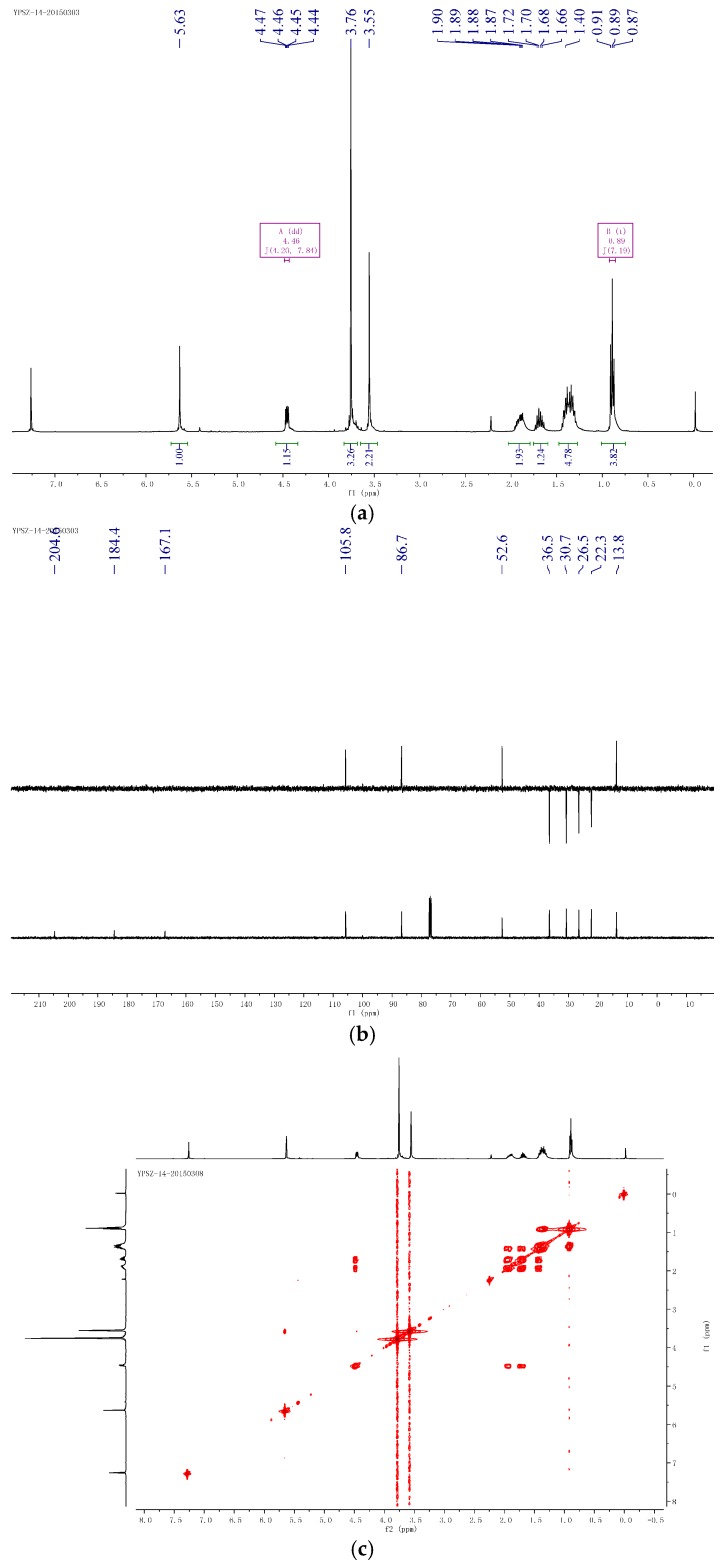

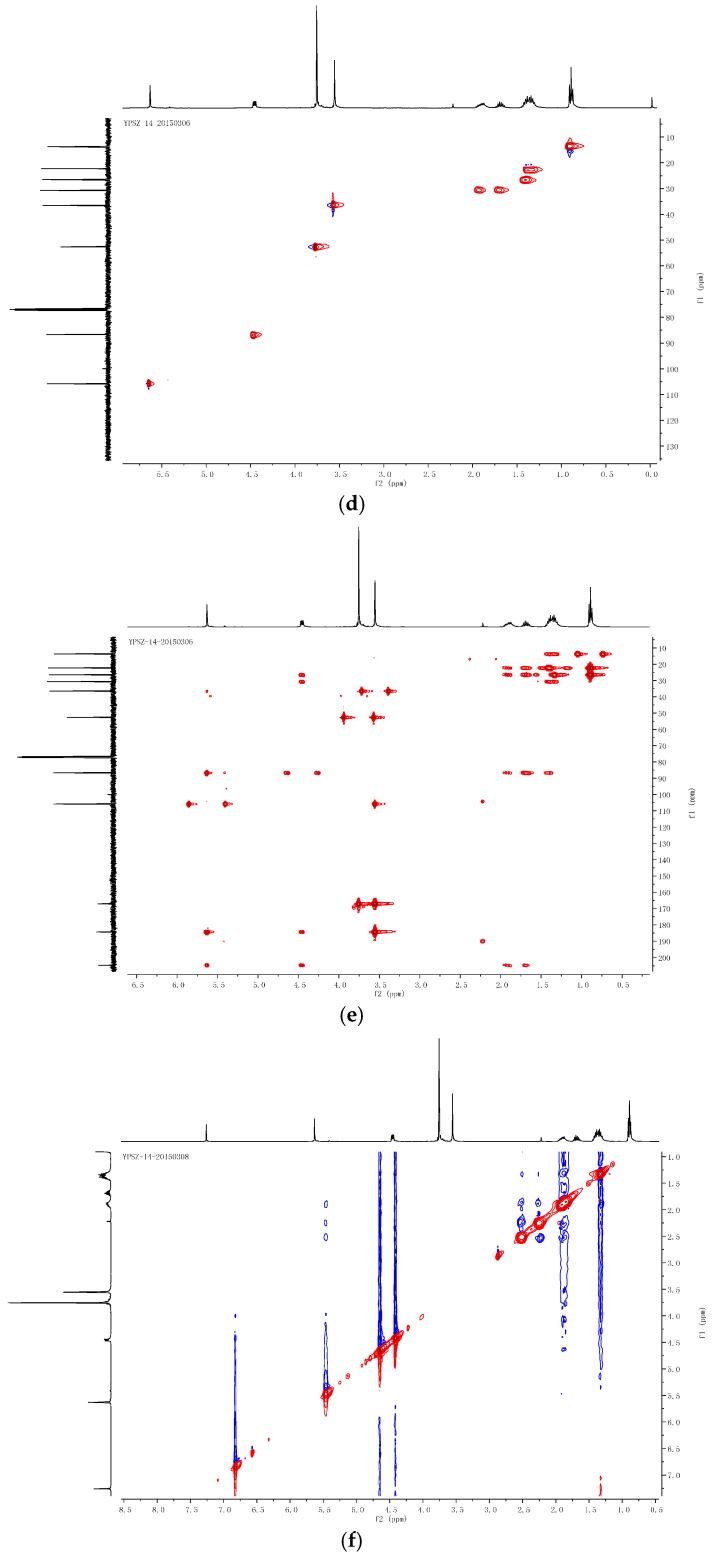
Supporting information of compound **2**. (**a**) ^1^H-NMR of compound **2**; (**b**) ^13^C-NMR and DEPT of compound **2**; (**c**) HSQC of compound **2**; (**d**) ^1^H-^1^H COSY of compound **2**; (**e**) HMBC of compound **2**; (**f**) NOESY of compound **2**.

Compound **2**, yellow oil (${\left[\alpha \right]}_{\text{D}}^{25}$ +82.0 (*c* 1.30, MEOH)), had the molecular formula of C_11_H_18_O_5_, as established by HRESIMS ion at m/z 253.1053 [M + Na]^+^ (cald. 253.1052) and ^13^C-NMR data. The IR spectrum exhibited absorption bands for hydroxyl groups (3416 cm^−1^) and carbonyl groups (1746 and 1699 cm^−1^). The ^1^H-NMR spectra exhibited signals for one methyl groups (H 0.89, 3H, t, *J* = 7.2 Hz), one oxymethyl (H 3.76, 3H, s), and a series of aliphatic methylene or methine multiplets (Table 1). The ^13^C-NMR spectrum, in combination with DEPT experiments, resolved 11 carbon resonances attributable to one ketone group (C 204.6), one carbonyl group (C 184.4), one tri-substituted double bond (C 167.1 and 105.8), two methyls, one sp^3^ oxygenated methines, and four sp^3^ methylenes (Table 1). As three degrees of unsaturation were consumed by one tri-substituted double bond, one kentone group, and one carbonyl group, then the structure of compound **2** should be a chain. The above-mentioned information was quite similar to that of co-isolated compound **4** reported from the same genus [12]. In comparison with compound **4**, the major differences of compound **2** were due to an additional ketone group and one carbonyl group indicating that compound **2** was a 2,6-hydrolysis derivative of compound **4**. HMBC correlations from H_3_CO-4 (H 3.76) to C-4 (C 167.1) assigned that the methoxyl group was linked with C-4 (Figure 2). The planar structure of **1** was further established by detailed interpretation of its 2D NMR data (Figure 5). The relative configuration of compound **2** was established by comparison of 1DNMR data with compound **4**. The absolute configuration of compound **4** was confirmed by chemical synthesis in previous work [12]. The absolute configuration of compound **2** was confirmed by the chemical transformation from **4** to **2** in methanol added with 1 equiv NaOH at 60 °C. Therefore, compound **2** was determined as (6*S*,2*E*)-6-hydroxy-3-methoxy-5-oxodec-2-enoic acid.

**Figure 6:**
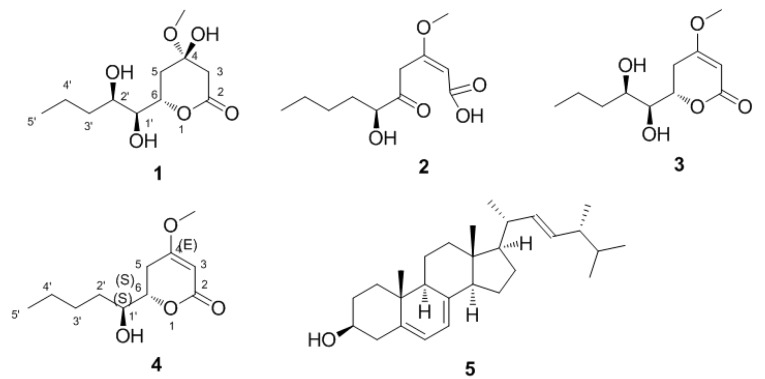
Structures of compounds **1**–**5**.

Based on the NMR and MS data, compounds **3**–**5** were identified as LL-P880γ [10], LL-P880α [13], and Ergosta-5,7,22-trien-3β-ol [14], respectively (Figure 6).

### 2.3. Antifungal Activity

As can be seen form the Table 2, among the five compounds, compounds **1**–**4** all showed notable anti-fungal activities with the minimal inhibitory concentration (MIC) values no more than 50 μg/mL for all the tested fungi; interestingly, compounds **1** and **2** possess the strong activities with the MIC values no more than 25 μg/mL. In addition, our present result also demonstrated that compound **5** possessed moderate anti-fungal effects on the four tested fungi (MIC values were higher than 200 μg/mL).

**Table 2:** Anti-fungal effects of compounds **1**–**5**.

	MIC (μg/mL)			
	*C. albicans*	*C. neoformans*	*T. rubrum*	*A. fumigatus*
**1**	6.25	3.13	25	25
**2**	12.5	12.5	6.25	3.13
**3**	12.5	50	50	50
**4**	6.25	3.13	50	25
**5**	>400	200	>400	>400
**KTZ**	0.0625	0.03125	0.5	1

Ketoconazole (KTZ) was used as positive control.

### 2.4. Cytotoxic Activity

From our present results showed in Table 3, compounds **1**–**4** showed significant cytotoxic activities on the MKN45, LOVO, A549 and HL-60 cancer cell lines with the IC_50_ values lower than 200 μM. In addition, the compounds **1**–**4** possessed notable cytotoxcities against human cancer cell lines of HL-60 cells with the IC_50_ values of below 100 μM. Besides, compounds **1**, **2**, **4** and **5** showed strong cytotoxities on the LOVO cell line with the IC_50_ values lower than 100 μM.

**Table 3:** Cytotoxic effects of compounds **1**–**5**.

	IC_50_ (μM)				
	MKN45	LOVO	A549	HepG2	HL-60
**1**	104.76 ± 5.34	50.97 ± 1.87	157.02 ± 2.01	>200	15.24 ± 0.34
**2**	135.87 ± 6.15	41.91 ± 1.07	>200	>200	30.09 ± 0.98
**3**	125.87 ± 5.76	139.96 ± 5.76	182.92 ± 5.98	>200	64.87 ± 1.47
**4**	65.28 ± 1.98	68.88 ± 2.98	125.79 ± 4.07	191.68 ± 6.94	30.75 ± 1.65
**5**	>200	65.20 ± 1.37	>200	>200	171.54 ± 4.97
**DOX**	0.14 ± 0.002	0.06 ± 0.002	0.16 ± 0.004	0.18 ± 0.004	0.01 ± 0.001

Doxorubicin (DOX) was used as positive control.

### 2.5. Discussion

In terms of orchid–fungus relationships research, most works are concerning about the functional role of mycorrhizal fungi on seed germination and plants survival [15]; relatively little is known of endophytic nature of plant aboveground tissues and their roles in the establishment and growth of epiphytic orchids [16]. In recent years, non-mycorrhizal endophytes have been recorded and recognized [17]. Some researches indicated that non-mycorrhizal endophytes may function a previously underestimated way [18]. However, knowledge of orchids including *Dendrobium* associated non-mycorrhizal fungi is limited. We have previously investigated endophytic fungi isolated from leaves, stems and roots of *D. officinale* attached to nine tree species in Yandang Mountain of Zhejiang, China (unpublished). In order to select meaningful fungi that can promote the growth and contents of the host, 134 endophytic fungal taxa were isolated co-cultured with sterile plantlets of *D. officinale* one by one. As a result, *Pestalotiopsis* sp. DO14 could significantly improve the main medicinal compounds contents (e.g., polysaccharides) of *D. officinale*, which is therefore selected for the present study to understand chemical constituents and pharmacological activities of its metabolites.

*Pestalotiopsis* (Amphisphaeriaceae) species are distributed widely in tropical and temperate ecosystems as saprobes, pathogens, and endophytes of living plants [19]. Species of *Pestalotiopsis* have become a topic of research in many microbial-chemical and pharmacological laboratories because they contain structurally complex, biologically active metabolites. Xu *et al.* [20] reviewed 160 different compounds isolated from species of Pestalotiopsis containing alkaloids, steroids, sesquiterpenes, triterpenes, coumarins, chromones, simple phenols, phenolic acids, lactones, *etc.* Antitumor, antifungal, and antimicrobial activities were the most notable bioactivities of secondary metabolites isolated from this genus [21]. However, to our knowledge, there have been no monoterpenoids reported from this genus.

Previous studies indicated that anti-fungal and antitumor are the two major activities of compounds isolated form endophytic fungus *Pestalotiopsis* sp. [21]. In addition, Chen *et al.* reported that 4-(3′,3′-Dimethylallyloxy)-5-methyl-6-methoxyphthalide (DMMP) isolated from the endophytic fungus *Pestalotiopsis* sp. possessed significantly antitumor effect via mitochondrial extrinsic apoptotic pathway [22]. Furthermore, induction of apoptosis is one of the important mechanisms of taxol isolated from the *Pestalotiopsis* sp. [4]. Previous investigations reported that the destruction of the bacterial cell might be the possible antifungal activity of compounds isolated from the *Pestalotiopsis* sp. [23,24]. In this study, we found that the endophytic fungus *Pestalotiopsis* sp. DO14 produced different varieties of metabolite classes that were not yet reported from *Pestalotiopsis* species and that showed potent cytotoxic and anti-fungal activities. Compounds **1** and **2** were new members of the monoterpenoids metabolites with strong cytotoxic and antifungal activities, and they represent the first isolation of monoterpenoids derivative from the genus *Pestalotiopsis*. Compound **3** was first isolated from *Penicillium* citreo-viride as a pestalotin analogue, which was a gibberellin synergist. Compound **4** was first isolated from *Penicillium* sp. We found they both have strong cytotoxic and antifungal activities in this study. Compound **5** was ergosterol derivative. Ergosterol is the precursor of vitamin D2 which is very important for human health. Ergosterol and its derivatives comprise a big family in mushrooms [25]. Compound **5** was isolated from *Pestalotiopsis* for the first time. However, more works are needed to be devoted to systemically investigate the potential mechanisms of antitumor and antifungal activities of these compounds isolated in our present research.

## 3. Experimental Section

### 3.1. Isolation and Identification of the Endophytic Fungus

Healthy shoots of *D. officinale* plants were collected in Yandang Mountain, Zhejiang Province, PR China. Samples were immediately placed in plastic bags, labeled, and taken to the laboratory store at 4 °C for isolation of endophytic fungi within 48 h of collection. The samples were washed and then cut into 3 cm-long segments before surface-sterilization. Shoot segments were surface sterilized by using the method of Wu *et al*. [26]. Then, the shoots was cut into 1 cm-long segments, and placed on potato dextrose agar (PDA) media containing 50 mg/L penicillin and incubated at room temperature for 14 days. The hyphal tip was transferred to new PDA plates and incubated at 26 °C until the pure mycelium covered most of the plate.

The isolated endophytic fungus DO14 was identified according to its morphology and ITS sequences by using the universal primers ITS5 and ITS4 following the reported protocol [27]. Obtained ITS sequence was compared by Blast search with reference sequences at the GenBank and all sequences were aligned with CLUSTAL X software [28]. The phylogenetic tree was performed using the neighbor-joining method. Identification of sequences was according to Wu *et al*. [27]. The sequence was submitted to GenBank (accession No. KP050569). The fungal strain DO14 was deposited in the China Center for Type Culture Collection (CCTCC) as CCTCC M 2015180.

### 3.2. Fermentation and Compounds Isolation

The fermentation extracts of DO14 were prepared as reported [29]. DO14 was inoculated in Erlenmeyer flasks with potato dextrose broth (PDB) and incubated on a rotary shaker (180 rpm) for 7 days at 28 °C. Crude fermentation broths were filtered and blended for extraction with ethyl ether. After extracted by ethyl ether, 6.9 g crude extracts were obtained, and, subsequently, the extracts were subjected to silica gel column chromatography, eluting with gradient petroleum ether-acetone (30:1–1:1). Combination of similar fractions by using TLC (Thin Layer Chromatography) analysis, seven fractions (A–G) were afforded. Then, fraction C was purified by gel filtration on Sephadex LH-20 to afford compound **5** (36 mg). Fraction E was subject to column chromatography over silica gel, Sephadex LH-20, and preparative TLC to afford 1 (23 mg), 2 (19 mg), 3 (27 mg). Compound **4** (28 mg) were isolated from the fractions F in the same way (Figure 1).

### 3.3. Antifungal Assay

Antifungal activities of the compounds **1**–**5** were assayed on the four common pathogens (available in the Chinese Academy of Sciences) follows: *Candida albicans*, *Cryptococcus neoformans*, *Trichophyton rubrum*, *Aspergillus fumigatus*. The MIC was used to evaluate anti-fungal activities of the isolated compounds **1**–**5**, and the MIC assay was carried out according to the previous reported method [29]. Briefly, the sabouraud dextrose agar was used for fungal culture. Dilutions of the compounds **1**–**5** were prepared as follows: 400, 200, 100, 50, 25, 12.5, 6.25, 3.13 and 1.56 μg/mL. In addition, ketoconazole (KTZ) was used as positive control, and the concentrations were prepared as follows: 8, 4, 2, 1, 0.5, 0.25, 0.125, 0.0625 and 0.03125 μg/mL. In addition, Dimethyl sulfoxide (DMSO) at a concentration of 1% was used to enhance the compounds’ solubility. Then, the pathogens (1–5 × 10^3^ CFU/mL) were seeded in the 96 well plates, and the total volume is 200 μL. MIC values were determined as the lowest samples’ concentrations that prevent visible fungal growth at 35 °C after 24 h, 72 h and 168 h of incubation for *Monilia*, *Cryptococcus* and hyphomycete, respectively [20,30].

### 3.4. Cytotoxic Assay

Cytotoxic assay was determined by using the MTT [3-(4,5-dimethylthiazole-2-yl)-2,5-diphenyltetrazoliumbromide] [31]. Briefly, five human cancer cell lines (available in the Chinese Academy of Sciences) were used: MKN45, LOVO, A549, HepG2, and HL-60. Briefly, cells with density of 1 × 10^5^ cells/mL were cultured in 96-well plates for 24 h with 10% FBS DMEM medium. Subsequently, cells were treated with test samples at a series nine concentrations (0.5, 1, 5, 10, 20, 40, 60, 80, and 100 μg/mL) for 24 h. Then, 20 μL MTT (5 mg/mL) was added for 4 h, and after removing the medium, 150 μL DMSO was added into each well to dissolve blue formazan crystals. Finally, the optical density (OD) values were read at a wavelength of 570 nm on a micro-plate reader (Labsystems, WellscanMR-2). Cell proliferation inhibition (%) was determined according to the results of MTT assay, and IC_50_ values of the compounds on MKN45, LOVO, A549, HepG2, and HL-60 cell lines were calculated by LOGIT method. Each experiment was repeated three times.

## 4. Conclusions

Our results indicate that compounds isolated from the DO14 could be valuable candidates as potent tumor inhibitors and be beneficial in the therapy of cancer diseases. Our study also underscores that endophytic fungi of *D. officinale* are great potential resources to discover novel agents for preventing or treating pathogens and tumors. However, further investigations are still needed to study the extraction process and pharmacological action mechanisms of the active constituents in metabolites of endophytic fungi isolated from *D. officinale*.
